# Data-informed deep optimization

**DOI:** 10.1371/journal.pone.0270191

**Published:** 2022-06-23

**Authors:** Lulu Zhang, Zhi-Qin John Xu, Yaoyu Zhang

**Affiliations:** 1 School of Mathematical Sciences, Institute of Natural Sciences, Shanghai Jiao Tong University, Shanghai, China; 2 MOE-LSC and Qing Yuan Research Institute, Shanghai Jiao Tong University, Shanghai, China; 3 Shanghai Center for Brain Science and Brain-Inspired Technology, Shanghai, China; Fuzhou University, CHINA

## Abstract

Motivated by the impressive success of deep learning in a wide range of scientific and industrial applications, we explore in this work the application of deep learning into a specific class of optimization problems lacking explicit formulas for both objective function and constraints. Such optimization problems exist in many design problems, e.g., rotor profile design, in which objective and constraint values are available only through experiment or simulation. They are especially challenging when design parameters are high-dimensional due to the curse of dimensionality. In this work, we propose a data-informed deep optimization (DiDo) approach emphasizing on the adaptive fitting of the the feasible region as follows. First, we propose a deep neural network (DNN) based adaptive fitting approach to learn an accurate DNN classifier of the feasible region. Second, we use the DNN classifier to efficiently sample feasible points and train a DNN surrogate of the objective function. Finally, we find optimal points of the DNN surrogate optimization problem by gradient descent. To demonstrate the effectiveness of our DiDo approach, we consider a practical design case in industry, in which our approach yields good solutions using limited size of training data. We further use a 100-dimension toy example to show the effectiveness of our approach for higher dimensional problems. Our results indicate that, by properly dealing with the difficulty in fitting the feasible region, a DNN-based method like our DiDo approach is flexible and promising for solving high-dimensional design problems with implicit objective and constraints.

## 1 Introduction

In recent years, deep learning has achieved impressive success not only in traditional artificial intelligence (AI) problems but also in many scientific and industrial applications [1]. More researchers realize that deep neural network (DNN) is a powerful tool to solve high-dimensional problems suffering from the “curse of dimensionality” (CoD). Currently, it has been a trend to actively explore the application of DNN to a wide range of scientific and industrial problems difficult to be solved by conventional methods [2]. For example, deep learning-based algorithms has been developed for solving high-dimensional PDEs [3, 4], stochastic control problems [5], robotic control problems [6, 7] as well as other applications. These successes motivate us to extend the application of DNN to a wider range of scientific and industrial problems. Note that, though DNN is a promising method for high dimensional problems, adapting it to specific problems and achieving success is still a highly nontrivial task. It is important not only to develop methods, but also to unravel the key issues encountered and their potential solutions in designing DNN-based algorithms.

In this paper, we explore the application of DNN in a specific class of optimization problems with implicit objective and constraints. This class of problems exhibit in many different scientific and industrial applications, such as modeling a biological neuronal network to meet a set of biological requirements on its dynamical performance in neuroscience [8], and optimizing a large set of design parameters to maximize the machine performance while satisfying physical constraints in industry [9, 10]. For such systems, the dependence between the model or design parameters and the corresponding performance often has no explicit formula, which restricts the application of optimization techniques in the complex industrial problem [11, 12]. Moreover, constraints of model/design parameters in these problems may also be very complex with no explicit formulas. Whether a set of model/design parameters is compatible to the constraints may only be examined through experiments or simulations. For convenience, we call such optimization problems *data-informed optimization problems*. Moreover, in contrary to traditional optimization problems which are often low-dimensional and can be analytically described and solved by many well-developed algorithms [13, 14], it is increasingly important to develop tractable approaches for high-dimensional data-informed optimization problems [15].

A viable method to solve a data-informed optimization problem is to use surrogate models to fit the objective and constraint functions. There are many conventional machine learning models which can be used to learn a surrogate function from discrete data [12, 16, 17]. Polynomial regression is a commonly used method since it is easy to use [18]. However, this parametric model has limited flexibility and generally does not fit well unless the true function which generates data has a similar form to the polynomial [19]. In general, it is mainly used in low-dimensional, linear or quadratic cases [15]. Another popular method called Kriging model has been developed for use in the fields of spatial statistics and geostatistics, and is especially popular in aerodynamic design [20, 21]. The Kriging method is based on Gaussian Processes and is sufficiently flexible to represent nonlinear and multimodal functions. However, Kriging model has many hyperparameters and it is untractable to tune them in high-dimensional problems [17, 22]. In addition, regular Kriging model can be correctly formulated only when the function to be approximated satisfies several assumptions of accuracy, smoothness, and continuity [23–25]. There are several other types of models often considered, such as radial basis functions (RBF) [26–28], multivariate adaptive regression splines(MARS) [19], wavelet modeling [29] and inductive learning [30]. Unfortunately, these conventional modeling techniques are mostly limited to lower dimensional problems due to the curse of dimensionality. Developing methods suitable for high dimensional problems is highly demanded [15].

In recent years, empirical and theoretical studies suggest that the DNN model, trained by gradient-based algorithms, can overcome the curse of dimensionality in fitting high-dimensional functions [31, 32]. It has also been observed in practice that the DNNs in general do not overfit even in an overparameterized setting without explicit regularizations [33]. A series of studies provide potential mechanisms underlying the non-overfitting puzzle of DNNs. For example, frequency principle, both in experiments and theory [34–37], shows that DNNs prefer to fit training data with low-frequency functions, which often leads to a good generalization performance due to the low frequency dominance in real data. From the optimization perspective, though training of a DNN surrogate is a highly non-convex problem [38], we empirically find that the first-order optimization method of gradient descent often converges efficiently and yields satisfying solutions. Moreover, using the well-developed platform like Tensorflow and PyTorch [39–41] implemented on GPUs, one can easily and efficiently train a DNN with even millions of parameters. Due to all these merits, deep learning emerges as an increasingly important surrogate model in exploring optimal solutions of practical problems in many fields, for example, robot manipulator control [42, 43], heat management [44, 45], industrial design and production [46].

Though our idea of using DNN surrogate for fitting objective and constraints from data looks straightforward and promising, the design of a DNN-based algorithm that works well in experiments is still nontrivial. We encounter the following difficulties. First, without an explicit feasible region, sampling feasible points well covering the feasible region is very difficult especially in a high-dimensional space. Usually, our prior knowledge of the feasible region gained from experience is in the form of a parameter box consisting of rough intervals for each parameter. However, as the dimension of parameter space gets higher, volume of the feasible region over the volume of even the smallest box containing it often vanishes exponentially fast [47]. We refer to this phenomenon the curse of dimensionality for sampling, which indicates that a random sampling in the box has almost no chance to obtain a feasible point. Second, the fitting of the feasible region itself similarly suffers from this curse of dimensionality for sampling in the sense that it is very difficult to obtain a balanced sample. Clearly, tackling above sampling difficulty is essential to the success of our algorithm design.

In this work, we propose the data-informed deep optimization (DiDo) approach to solve potentially high-dimensional complex optimization problems with implicit objective and constraints. Our approach emphasizes the DNN-based adaptive fitting of the feasible region which can overcome the curse of dimensionality for sampling. The idea is as follows. Starting from a small size of initial samples, we train a coarse DNN classifier for the feasible region identification. Then, at each iteration, we sample an additional set of points informative to the improvement of the last DNN classifier, based on which we further update the DNN classifier to improve its accuracy. With an accurate DNN classifier obtained after a few iterations, we can easily use Langevin Monte Carlo sampling to generate feasible samples well covering the feasible region, by which an accurate DNN surrogate for the objective function can be fitted. Finally, with accurate DNN surrogates for both objective and constraints, we can easily implement conventional gradient-based optimization techniques to find candidates of optimal parameters.

As for the application, our DiDo approach can be applied to solve a series of engineering design problems, in which performance of the designed products is evaluated through simulation and the design parameters are constrained in an implicit region, e.g. determined by complex geometric constraints. For demonstration, we first consider a specific problem in industry, which is to optimize the 6-dimensional design parameters for the rotor profile of double screw compressor to maximize the actual flow. Without the need of carefully adjusting hyper-parameters, the best actual flow found by our DiDo approach is much better than the result obtained by original hand-craft approach. To illustrate the effectiveness of DiDo approach in higher dimensional problems, we consider a 100-dimension toy example. The optimal value found by our approach is far beyond the optimal value in training samples, and is close to the true optimal value. These results demonstrate that our DiDo approach can indeed solve high-dimensional data-informed optimization problems.

The main contribution of our work is outlined as follows:

1. We explore the application of DNN by proposing the DiDo approach to solve a specific class of optimization problems with implicit constraints and objective function. Importantly, we identify the curse of dimensionality for sampling as the main challenge for the design of a DNN-based algorithm for these problems.
2. We propose the DNN-based adaptive fitting method for fitting the feasible region, which overcomes the curse of dimensionality for sampling and greatly improves sampling efficiency as demonstrated by numerical experiments.
3. We combine an accurate DNN-based classifier for the feasible region with Langevin Monte Carlo (LMC) sampling to efficiently generate feasible samples well covering the feasible region, which is key to obtain an accurate DNN surrogate of the objective function.

The rest of paper is organized as follows. Initially, we give a brief preliminary about the notation, DNN and LMC. Followed by the main contents of this paper, our data-informed deep optimization approach, that is, using a deep-based method to solve a type of optimization problem which is different from the traditional ones. Then for demonstrating our DiDo approach, a practical design case in industry and a 100-dimensional toy example are shown in detail. Finally, we make a conclusion and discuss the future work.

## 2 Preliminary

### 2.1 Notation

In this paper, we use the following notations, see Table 1.

**Table 1:** Notation.

*x*	scalar component of ***x***
***x***	optimization variable
*d*	dimension of optimization variable
[*n*]	index set {1, 2, …, *n*}
*f*(***x***)	objective function
Ω	feasible region determined by the considered problem
∂Ω	the true boundary of implicit feasible region
*I*_Ω_(***x***)	indicator function of the region Ω, i.e., if ***x*** ∈ Ω, *I*_Ω_(***x***) = 1; otherwise, *I*_Ω_(***x***) = 0
$D_{\text{obj}}=\{{\left(x_{i};f\left(x_{i}\right)\right\}}_{i=1}^{n_{o}}$	training set for DNN fitting
$D_{c}={\left\{\left(x_{i};I_{\Omega }\left(x_{i}\right)\right)\right\}}_{i=1}^{n_{c}}$	training set for DNN classifier
$f_{\theta_{\text{o}}}\left(x\right)$	DNN surrogate model for objective function
$f_{\theta_{c}}\left(x\right)$	DNN classifier neural network for feasible region

### 2.2 DNN

The general setup for a DNN is reviewed as follows. A fully connected DNN of *H* layers is denoted by

$$\begin{array}{cc} & f_{\theta }\left(x\right)=W^{\left[H-1\right]}\sigma \text{o}\left(\cdots \left(W^{\left[1\right]}\sigma \text{o}\left(W^{\left[0\right]}\sigma \text{o}+b^{\left[0\right]}\right)+b^{\left[1\right]}\right)\cdots \right)+b^{\left[H-1\right]}, \end{array}$$

where $x\in R^{d}$, $W^{\left[l\right]}\in R^{m_{l-1}\times m_{l}}$, $b^{\left[l\right]}\in R^{m_{l-1}}$, *m*_0_ = *d*, *m*_*H*_ = 1, *σ* is the activation function and “o” means entry-wise operation. The set of parameters for DNN is denoted by

$$\begin{array}{c} \theta =(W^{\left[0\right]},W^{\left[1\right]},\cdots ,W^{\left[H-1\right]},b^{\left[0\right]},b^{\left[1\right]},\cdots ,b^{\left[H-1\right]}), \end{array}$$

For the regression problem of fitting a training set ${\left\{\left(x_{i},y_{i}\right)\right\}}_{i=1}^{n}$, where $x_{i}\in R^{d}$ and $y_{i}\in R$ for each *i*, the commonly used loss functions are mean-square error (MSE), that is,

$$\begin{array}{c} L\left(\theta \right)=\frac{1}{n}\sum_{i=1}^{n}{\left(f_{\theta }\left(x_{i}\right)-y_{i}\right)}^{2}, \end{array}$$

and root-mean-square error (RMSE), that is,

$$\begin{array}{c} L\left(\theta \right)=\sqrt{\frac{1}{n}\sum_{i=1}^{n}{\left(f_{\theta }\left(x_{i}\right)-y_{i}\right)}^{2}}. \end{array}$$

We use MSE when training DNN to fit the objective function. and use RMSE to measure the training error and test error of the DNN.

For the classification problem of fitting a training set ${\left\{\left(x_{i},q_{i}\right)\right\}}_{i=1}^{n}$, where $x_{i}\in R^{d}$ and *q*_*i*_ ∈ {0, 1} for each *i*, the loss function we used is binary-cross-entropy (BCE), that is,

$$\begin{array}{c} L\left(\theta \right)=-\frac{1}{n}\sum_{i=1}^{n}\left[q_{i}logf_{\theta }\left(x_{i}\right)+\left(1-q_{i}\right)log\left(1-f_{\theta }\left(x_{i}\right)\right)\right] \end{array}$$

In cases given in this paper, the activation function is fixed to GELU function. GELU is a smooth non-saturating activation function that can alleviate gradient vanishing. Empirically, a GELU DNN is efficient to train and generalizes well for smooth problems we considered. Note that one can also consider other smooth non-saturating activation functions like Swish, ELU and SELU to achieve similar training and generalization performance.

During the training of neural network, the parameters of the DNN in each epoch are updated by a gradient-based optimization algorithm, e.g. gradient descent (GD), stochastic gradient descent (SGD) or Adam. To speed up the training process, we update the parameters of DNN using Adam [48].

### 2.3 Langevin Monte Carlo (LMC)

There are many mature methods to sample data from a desired probability distribution, such as Markov chain Monte Carlo, Metropolis-Hastings, Hamiltonian Monto Carlo and Split Monte Carlo. For convenience, in our experiments, we use overdamped Langevin Monte Carlo (LMC).

LMC is a common method to sample data following a Boltzmann distribution [49–52]. This method is based on evolving a stochastic differential equation (SDE), that is,

$$\begin{array}{c} dx=-\nabla E\left(x\right)dt+\sqrt{\frac{2}{\beta }}dW, \end{array}$$

where *β* is positive hyperparamter and *W* is the Brownian motion. The steady-state distribution of this SDE is proportional to *e*^−*βE*(***x***)^ and it satisfies the detailed balance condition. The set of long-time solution of the SDE follows the Boltzmann distribution ∼*e*^−*βE*(***x***)^. We use the first-order Euler-Maruyama scheme to solve the SDE, i.e. each data point ***x*** is updated according to $x^{t+1}=x^{t}-\alpha \nabla E\left(x^{t}\right)+\sqrt{\frac{2\alpha }{\beta }}\xi^{t}$, where *ξ*^*t*^ ∼ *N*(0_*d*_, *I*_*d*_).

Note that we choose appropriate energy function *E*(***x***) for different tasks. In our experiment, we use $E\left(x\right)={\left(f_{\theta_{c}}\left(x\right)-0.5\right)}^{2}$ to sample data concentrated around the boundary of the predicted-feasible region for DNN classifier $f_{\theta_{c}}\left(x\right)$ and use $E\left(x\right)={\left(f_{\theta_{c}}\left(x\right)-1\right)}^{2}$ to efficiently sample more feasible data, where $f_{\theta_{c}}\left(x\right)$ denotes the DNN classifier.

A simple version of LMC method is shown in algorithm 1.

**Algorithm 1**: Langevin Monte Carlo (LMC)

**Data**: *T*: total iteration steps; energy function *E*(***x***); step length *α*; positive *β*; $X^{0}={\left\{x_{i}^{0}\right\}}_{i=1}^{n}$: initial data set.

**Result**: *X*^*T*^

1 **for** *t* = 0 **to** *T* **do**

2  $x_{i}^{t+1}=x_{i}^{t}-\alpha \nabla E\left(x_{i}^{t}\right)+\sqrt{\frac{2\alpha }{\beta }}\xi_{i}^{t}$, where $\xi_{i}^{t}∼N\left(0_{d},I_{d}\right),i\in \left[n\right]$;

3 **end**

4 Get $X^{T}={\left\{x_{i}^{T}\right\}}_{i=1}^{n}$ and $X^{T}∼e^{-\beta E(x)}$

## 3 Data-informed deep optimization

In this section, we introduce the framework of Data-informed deep optimization (DiDo) approach for solving high-dimensional optimization problems, in which the objective function and the constraints are only available through samples without explicit formula. The DiDo approach shows an indispensable value beyond the tradition optimization approach in high-dimensional data-informed problems.

### 3.1 Data-informed problem formulation

The data-informed optimization problem is formulated as follows.

**Data-informed optimization problem**:

$$\begin{array}{c} \min_{x\in \Omega }\;f\left(x\right), \end{array}$$ (1)

where objective function *f*(***x***) and feasible region Ω are implicit which can only be evaluated through simulation at certain sampling points. In practice, Ω is often defined by a series of implicit constraints as Ω = {***x***|*f*_*i*_(***x***) ≤ 0, *i* = 1, 2, …, *L*}.

### 3.2 Deep optimization approach

**Fig 1:**
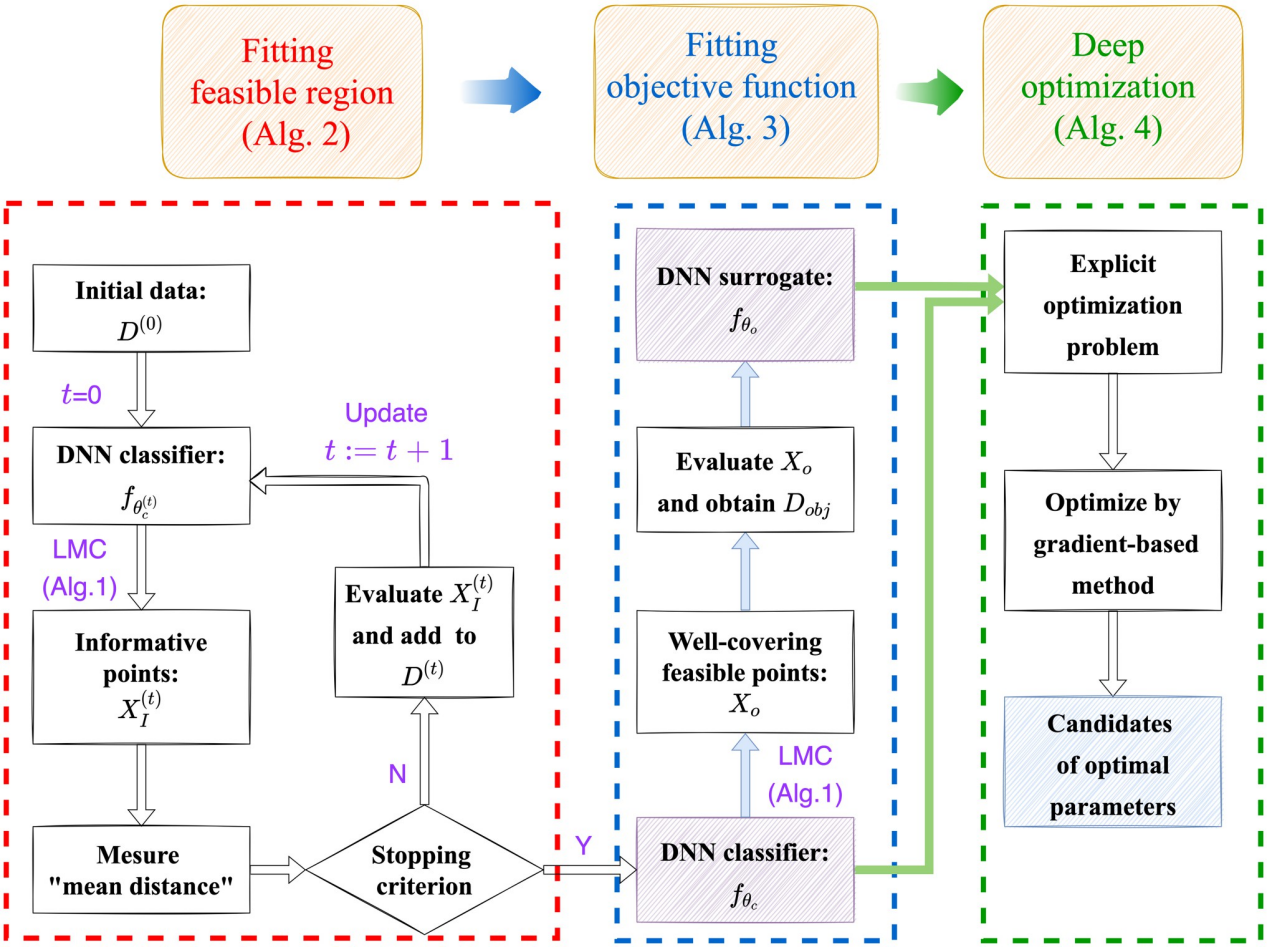
The flow chart of the DiDo approach.

We propose a deep optimization approach to solve the data-informed optimization problem (see Fig 1 for a flow chart). Our general idea to deal with a problem with implicit objective function and feasible region is to fit them by DNN surrogates from data. Then we can optimize this problem with common gradient-based method. Note that the training of the objective function relies on an explicit and accurate surrogate of feasible region for generating high quality training samples well covering the whole feasible region. Therefore, we first train a DNN classifier $f_{\theta_{c}}\left(x\right)$ through an iterative process from an initial sample set. Then we use $f_{\theta_{c}}\left(x\right)$ to generate random samples from feasible region and evaluate the corresponding values of the objective function. Then we build the training data set $D_{\text{obj}}={\left\{x_{i},f\left(x_{i}\right)\right\}}_{i=0}^{n_{o}}$ by simulation, whose inputs are sampled randomly from feasible region based on $f_{\theta_{c}}\left(x\right)$. Through fitting *D*_obj_, we obtain a DNN $f_{\theta_{\text{o}}}\left(x\right)$ as a surrogate of the objective function *f*(***x***). Finally, by optimizing $f_{\theta_{\text{o}}}\left(x\right)$ with surrogate constraints $f_{\theta_{c}}\left(x\right)\geq 0.5$ ($f_{\theta_{c}}\left(x\right)=0.5$ is regarded as surrogate boundary), we can get candidates of the optimal parameters of the problem (1), which should be close to the true optimal parameters of the problem.

#### 3.2.1 Fitting feasible region

Generally, without an explicit feasible region, it is difficult to generate well distributed feasible training samples especially in a high-dimensional problem. With a blind sampling, training samples are likely far from the decision boundary, i.e., boundary of the feasible region, resulting in an inaccurate fitting of the DNN classifier. To overcome this difficulty in our deep optimization approach, we propose a DNN-based adaptive fitting approach which adds new samples around the boundary of current DNN classifier and retrain it at each iteration. Using this approach, we can efficiently obtain an accurate DNN classifier $f_{\theta_{c}}\left(x\right)\in \left[0,1\right]$ of the feasible region through several rounds of iteration.

Initially, we uniformly sample $X_{I}^{\text{ini}}$ in a selected region *B* based on the prior knowledge of the considered problem and train the classifier $f_{\theta_{\text{c}}^{\left(0\right)}}\left(x\right)$ by $D^{\left(0\right)}=\left\{\left(x_{i},I_{\Omega }\left(x_{i}\right)\right)|x_{i}\in X_{I}^{\text{ini}}\right\}$. Empirically balancing the feasible and infeasible points benefits the performance of the classifier. Note that many problems whose optimal parameters close to the boundary of the feasible region require highly accurate DNN classifier (see example in Fig 6). We propose a DNN-based adaptive fitting approach to efficiently improve the accuracy of classifier $f_{\theta_{c}^{\left(t\right)}}\left(x\right)$ at each iteration step *t*. For classification problem, generally, the points close to the decision boundary is of crucial importance to determine the classifier, e.g., support vectors for support vector machine (SVM). Therefore, at each iteration step, we add new training data sampled near the decision boundary of classifier $f_{\theta_{c}^{\left(t\right)}}\left(x\right)$ by LMC method (see algorithm 1 for details) and train a new classifier $f_{\theta_{c}^{\left(t+1\right)}}\left(x\right)$ initialized by $\theta_{c}^{\left(t\right)}$.

**Fig 2:**
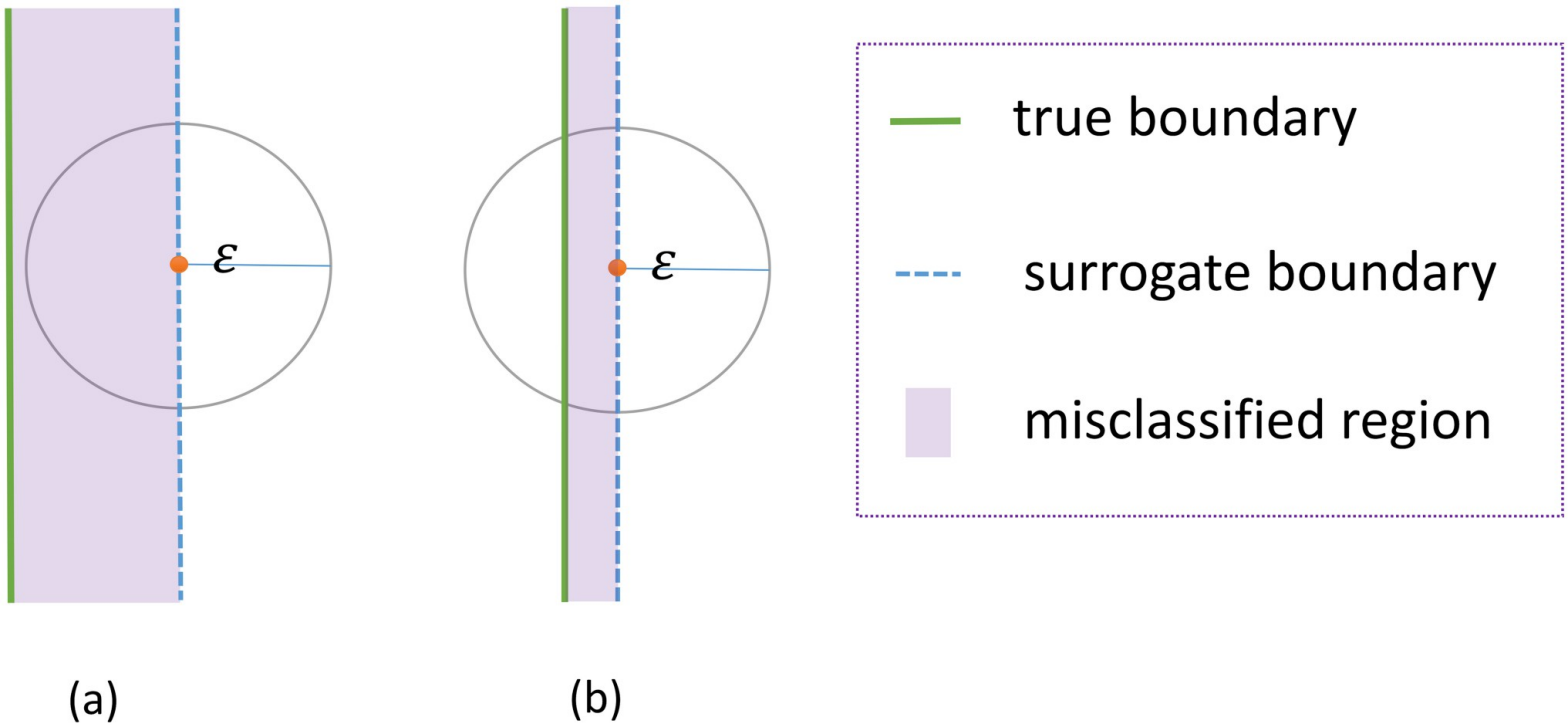
Illustration of “mean distance” for stopping criterion. Illustration of the relation between prediction accuracy and the “mean distance”. Intuitively, we can use predicted accuracy on perturbed points to quantify the quality of the classifier. (a) For red point on the surrogate boundary, the distance to the true boundary is larger than *ϵ*, and the prediction accuracy is roughly 50%; (b) for red point on the surrogate boundary, the distance to the true boundary is much smaller than *ϵ*, and the prediction accuracy is close to 1.

For a stopping criterion, it is crucial to determine whether the surrogate boundary is close to the true boundary, e.g., their “mean distance” is smaller than certain tolerance *ϵ*. Intuitively, for any point on the surrogate boundary, if its distance to the true boundary is larger than the *ϵ*, then the prediction accuracy of the DNN classifier in the *ϵ*-neighborhood of this point is roughly 50% (see Fig 2(a) for illustration); otherwise, if the distance is much smaller than *ϵ*, then the prediction accuracy in the *ϵ*-neighborhood should be close to 1 (see Fig 2(b) for illustration). Therefore, we sample some points close to the surrogate boundary by LMC method (see algorithm 1 for details) and perturbed them by Gaussian noise of covariance matrix *σ*^2^ *I*_*d*_, where *σ* is roughly *ϵ* due to concentration in the equator [47]. When the predicted accuracy of the classifier on these points is higher than a expected value, say 95%, we stop the iteration.

The detail of our DNN-based adaptive fitting approach is shown in algorithm 2.

**Algorithm 2**: DNN-based adaptive fitting approach

**Data**: *B*: region of initial sampling depending on the considered problem; *n*_0_: initial sample size; *n*_1_: adding sample size at each iteration; $E_{t}\left(x\right)={\left(f_{\theta_{c}^{\left(t\right)}}\left(x\right)-0.5\right)}^{2}$: energy function used in LMC method; *σ*: standard deviation of noise term; *β*: positive hyperparameter used in LMC method.

**Result**: Good classifier: $f_{\theta_{c}}\left(x\right)$

1 Uniformly sample $X_{I}^{\text{ini}}$ in *B*;

2 Define $D^{\left(0\right)}=\left\{\left(x_{i},I_{\Omega }\left(x_{i}\right)\right)|x_{i}\in X_{I}^{\text{ini}},i\in \left[n_{0}\right]\right\}$;

3 Define *t* = 0;

4 **do**

5  Train $\theta_{c}^{\left(t\right)}$ of $f_{\theta_{c}^{\left(t\right)}}\left(x\right)$ by *D*^(*t*)^ with Adam;

6  Use LMC method with proper initialization and *E*_*t*_(***x***) to sample *n*_1_ data following distribution $\sim e^{-\beta E_{t}(x)}$ and obtain input set $X_{I}^{\left(t\right)}$;

7  Perturbation: $X_{P}^{\left(t\right)}=\left\{x+\xi |x\in X_{I}^{\left(t\right)},\xi ∼N\left(0_{d},\sigma^{2}I_{d}\right)\right\}$;

8  Evaluate the classification accuracy *acc* of $f_{\theta_{c}^{\left(t\right)}}\left(x\right)$ on $X_{P}^{\left(t\right)}$

9  **if** *acc* ≥ 95% **then**

10   $f_{\theta_{c}}\left(x\right)\leftarrow f_{\theta_{c}^{\left(t\right)}}\left(x\right)$;

11   break;

12  **end**

13  Evaluate $X_{I}^{\left(t\right)}$ and add to the training data $D^{\left(t+1\right)}=\left\{\left(x_{i},I_{\Omega }\left(x_{i}\right)\right)|x_{i}\in X_{I}^{\left(t\right)}\right\}\cup D^{\left(t\right)}$;

14  Update *t* ← *t* + 1;

15 **while**;

#### 3.2.2 Fitting objective function

For a high-dimensional large-scale problem, with implicit boundary, it is difficult to efficiently sample diverse training data. However, with explicit classifier obtained above, we can use LMC with energy function $E\left(x\right)={\left(f_{\theta_{c}}\left(x\right)-1\right)}^{2}$ to generate high quality training samples *D*_obj_ = {(***x***_*i*_, *f*(***x***_*i*_)} well covering the feasible region of considered problem. By training the DNN by *D*_obj_, we can get the DNN surrogate $f_{\theta_{\text{o}}}\left(x\right)$ of the objective function.

The detail of fitting objective function is shown in algorithm 3.

**Algorithm 3**: Fitting of objective function

**Data**: classifier $f_{\theta_{c}}\left(x\right)$; a non-empty feasible set of $f_{\theta_{c}}\left(x\right)$: $X_{S}^{\text{ini}}$; a large enough number *n*_*t*_; energy function $E\left(x\right)={\left(f_{\theta_{c}}\left(x\right)-1\right)}^{2}$.

**Result**: DNN surrogate $f_{\theta_{\text{o}}}\left(x\right)$

1 Generate initial points for LMC method: $X_{0}=\left\{x_{i}|x_{i}\in X_{S}^{\text{ini}},i\in \left[n_{t}\right]\right\}$

2 Use LMC method with *E*(***x***) to sample data following distribution ∼*e*^−*βE*(***x***)^ and obtain $X_{S}^{T}$

3 Select data in the feasible region of real system Ω: $X_{\text{o}}=\Omega \cap X_{S}^{T}$;

4 Obtain training data for the objective function: *D*_obj_ = {(***x***_*i*_, *f*(***x***_*i*_))|***x***_*i*_ ∈ *X*_*o*_, *i* ∈ [*n*_*t*′_]};

5 Train ***θ***_o_ of $f_{\theta_{\text{o}}}\left(x\right)$ by *D*_obj_ with Adam.

### 3.3 Deep optimization

Based on the accurate DNN surrogate models of constraints and objective function obtained above, the data-informed optimization problem (1) turns to be the following explicit optimization problem,

$$\begin{array}{c} \underset{x}{\text{min}}\;f_{\theta_{\text{o}}}\left(x\right)\\ \;s.t.\;0.5-f_{\theta_{c}}\left(x\right)\leq 0, \end{array}$$ (2)

where 0.5 is the threshold of the DNN classifier $f_{\theta_{c}}\left(x\right)\in \left[0,1\right]$ for prediction.

The problem (2) is a conventional optimization problem with constraints. To solve it, we first rewrite it as an unconstrained problem, making the inequality constraint implicit in the objective

$$\begin{array}{c} \underset{x}{\text{min}}\;f_{\theta_{\text{o}}}\left(x\right)+I_{-}\left(0.5-f_{\theta_{c}}\left(x\right)\right), \end{array}$$

where $I_{-}:R\mapsto R$ is the indicator function for the non-positive real number,

$$\begin{array}{c} I_{-}\left(u\right)=\{\begin{array}{cc} 0, & \text{if}\;u\leq 0;\\ \infty , & \text{if}\;u>0.\; \end{array} \end{array}$$

However, the indicator function *I*_−_ is not differentiable. We approximate the indicator function *I*_−_ by a “soft” function. For example, we use the interior-point method. The basic idea of interior-point method is to approximate the indicator function *I*_−_(*u*) by the barrier function and a common barrier function is logarithmic barrier, $-\left(\frac{1}{t}\right)log\left(-u\right)$, where *t* > 0 is a hyperparameter that sets the accuracy of the approximation [53].

Substituting *I*_−_(*u*) with $-\frac{1}{t}log\left(-u\right)$ gives the approximation

$$\begin{array}{c} \underset{x}{\text{min}}\;f_{\theta_{\text{o}}}\left(x\right)-\left(\frac{1}{t}\right)log\left(-\left(0.5-f_{\theta_{c}}\left(x\right)\right)\right). \end{array}$$ (3)

To solve problem (3), we use gradient descent (GD) for convenience. Although simple, we find that GD is often an effective optimization algorithm in DiDo.

The deep optimization is concluded in algorithm 4.

**Algorithm 4**: Deep optimization

**Data**: $f_{\theta_{c}}\left(x\right)$: well-trained DNN classifier; $f_{\theta_{\text{o}}}\left(x\right)$: DNN surrogate model for fitting objective function.

**Result**: Candidates of optimal parameters

1 Substitute $f_{\theta_{\text{o}}}\left(x\right)$ and $f_{\theta_{c}}\left(x\right)$ into problem (3);

2 Solve problem (3) by gradient-descent-based optimization algorithms, such as gradient descent (GD);

3 Get candidates of optimal parameters of the problem (1).

The proposed methodology gives a schematic process to search for candidates of optimal parameters (see Fig 1) for high dimensional optimization problem with implicit feasible region and objective function. As we will show, it is well suitable for data-driven inferences using deep neural networks which can efficiently differentiate.

Remark that even when we can analytically characterize the feasible region by a set of equations, we can also train a DNN surrogate to represent the feasible region. In such case, our approach can still bring benefits, for example, using DNN classifier can soft the boundary of the feasible region and we can easily determine the normal vector of the boundary.

## 4 Optimal rotor profile design

In this section, we apply the DiDo approach to solve an engineering design problem to show its effectiveness.

### 4.1 Problem description

Screw compressor is widely used in refrigeration, mining, petrochemical and other industries because of its high reliability, good power balance, less leakage and high efficiency. As the core component of twin-screw compressor, optimizing the design of rotor profile would vastly benefit the mechanical performance of the screw compressor. The rotor profile is smoothly connected by several arcs and arc envelopes together. Empirically, we can parameterize the rotor profile by 6 parameters, $x=\left[r,r_{3},r_{\text{o}},r_{\text{o}2},u_{1},R\right]\in R^{6}$, where *r*, *r*_3_, *r*_o_, *r*_o2_, *R* are radius of the arc and *u*_1_ is an angle [54, 55]. Then, the optimization of the rotor profile becomes an optimization problem w.r.t. the 6 parameters.

In our example, the performance of a design parameter set, consisting of the 6 design parameters, is measured by the actual flow of the rotor, which is an important performance indicator for large compressor, through computational fluid dynamics simulation. Our goal is to find a rotor profile that can maximize the actual flow.

Remark that not all parameters in $R^{6}$ are feasible for the design. They should satisfy a set of implicit constraints related to geometrical properties of the rotor. Therefore, both the objective and the constraint functions are data-informed, i.e., they are only available on a set of data points through simulation. In the following, we demonstrate the effectiveness of our DiDo approach on this problem.

### 4.2 Feasible region learned by a DNN classifier

In this example, we first use the DNN-based adaptive fitting approach in algorithm 2 to train the DNN classifier $f_{\theta_{c}}\left(x\right)$, which is a fully connected DNN with hidden layer sizes 800-600-400-200 equipped with a sigmoid function at the output layer. Without loss of generality, we choose 0.5 as threshold to determine the surrogate feasible region, i.e., $f_{\theta_{c}}\left(x\right)\geq 0.5$.

Remark that, we carefully choose the initial sample region *B*, such that the number of feasible points and non-feasible points are balanced in the initial training data. For the effectiveness of DNN training, we normalize each parameter to a mean zero and variance one input variable.

We set initial sample size *n*_0_ = 8000 and we set *n*_1_ = 5000 samples in each iteration. With algorithm 2, we can obtain a well-trained classifier $f_{\theta_{c}}\left(x\right)$.

**Fig 3:**
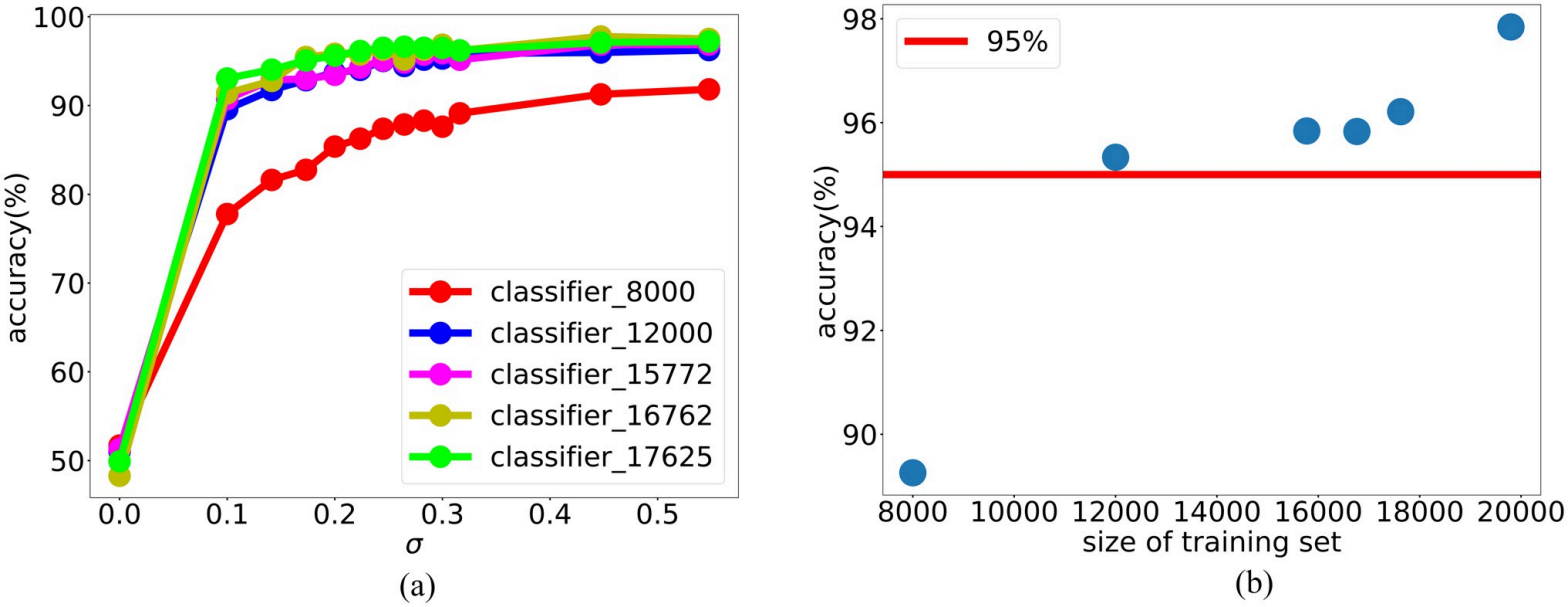
The improvement of DNN classifier through adaptive fitting. (a) Classification accuracy of the DNN classifier on the perturbed terms during iteration. Note that, at each iteration *t*, we apply an extra constraint $|f_{\theta_{c}}^{\left(t\right)}\left(x_{i}\right)-0.5\left|\leq 0.1\right\}$ to the points sampled by LMC. In the two figures, label accuracy means classification accuracy after perturbation. As we add more data, the magnitude of the perturbed term when classifier accuracy on perturbed term achieve 100% gets smaller, which means the performance of classifier is better. (b) Classification accuracy of the DNN classifier on the fixed standard deviation of the perturbed terms, where variance *σ*^2^ = 0.1. The classification accuracy is getting better as we update the DNN classifier.

To show effectiveness of the DNN-based adaptive fitting approach, we show the accuracy of the DNN classifier on the samples at surrogate boundary at each iteration with Gaussian noise perturbation during the iteration. As shown in Fig 3(a), for each curve, which is the accuracy of the classifier w.r.t. different noise standard deviation, as the perturbation noise increases, the accuracy increases. This indicates that the classifier is more accurate on the samples that deviate more from the boundary, which provides a rationale for our DNN-based adaptive fitting approach focusing on training the boundary. Compared with different iterations, indicated by different colored curves, as the iteration proceeds accompanied by the increasing of training samples, the classifier is improved. For example, as shown in Fig 3(b), considering a fixed noise with variance 0.1, the accuracy of the classifier almost monotonically increases as the size of the training set.

### 4.3 Objective function learned by a DNN model

We use a DNN surrogate to fit the objective function, i.e. a mapping from a designed rotor profile to the actual flow. By algorithm 3, we use the classifier $f_{\theta_{c}}\left(x\right)$ obtained above to generate a training set *D*_obj_ of size *n*_*t*′_ = 500 and train a GELU-DNN $f_{\theta_{\text{o}}}\left(x\right)$ of hidden layer size 1024-512-256-128. Remark that in algorithm 3, the ratio of the data in true feasible region to the data obtained by LMC is close to 1. The test accuracy of the DNN $f_{\theta_{\text{o}}}\left(x\right)$ is evaluated on a test data set consisting of 2000 samples.

**Fig 4:**
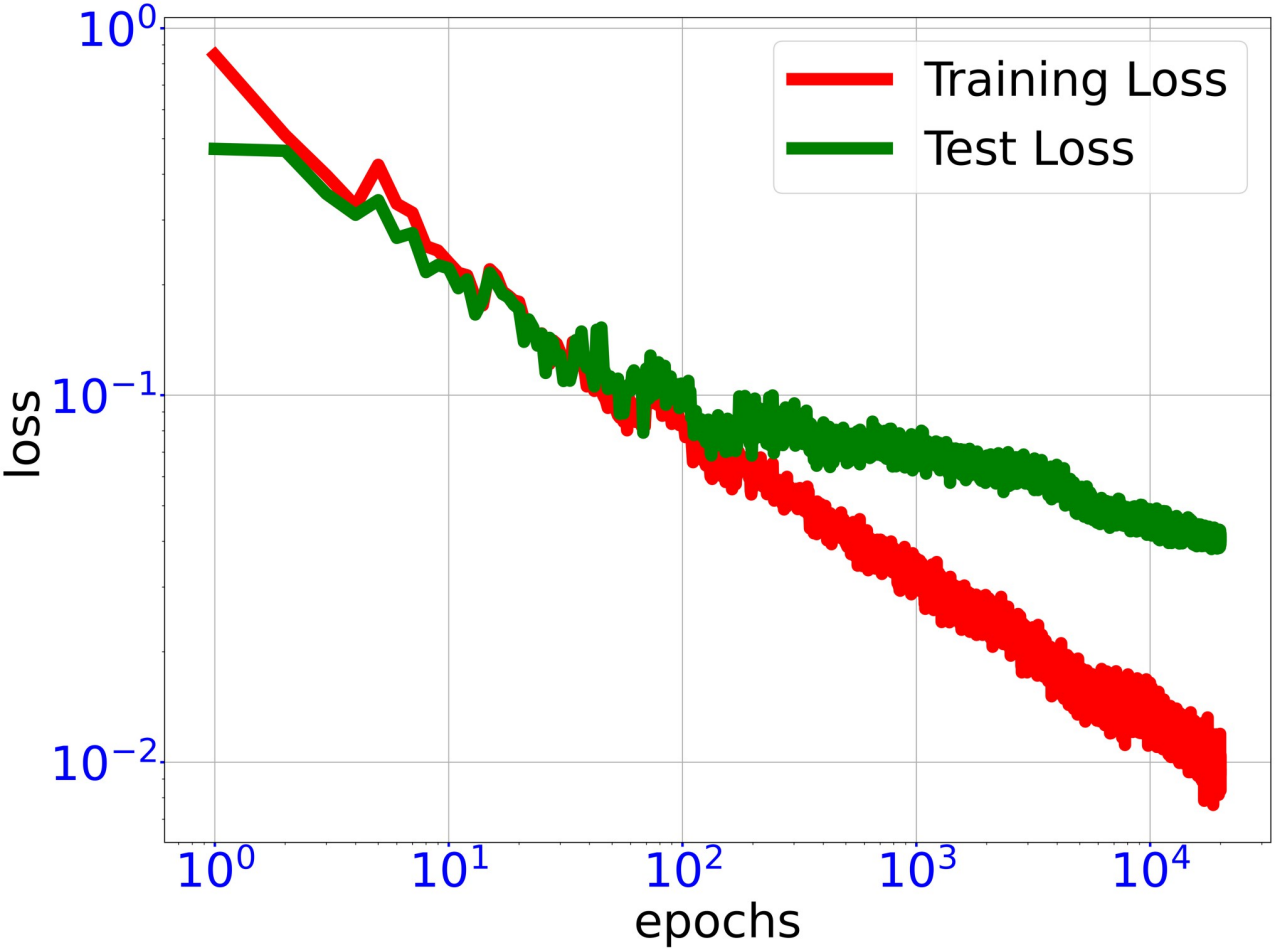
Training trajectory of DNN loss functions. Trajectory of training loss and test loss through training DNN surrogate $f_{\theta_{\text{o}}}\left(x\right)$ for fitting objective function of optimal rotor profile problem. In the end of training, the test loss is significantly larger than the training loss, indicating the DNN training is close to convergence.

As shown in Fig 4, after training, the normalized RMSE training error is ∼0.01 whereas the normalized RMSE test error is ∼0.04.

### 4.4 Deep optimization

Then we solve the problem with data-informed deep optimization approach in algorithm 4 using $f_{\theta_{\text{o}}}\left(x\right)$ and $f_{\theta_{c}}\left(x\right)$.

**Fig 5:**
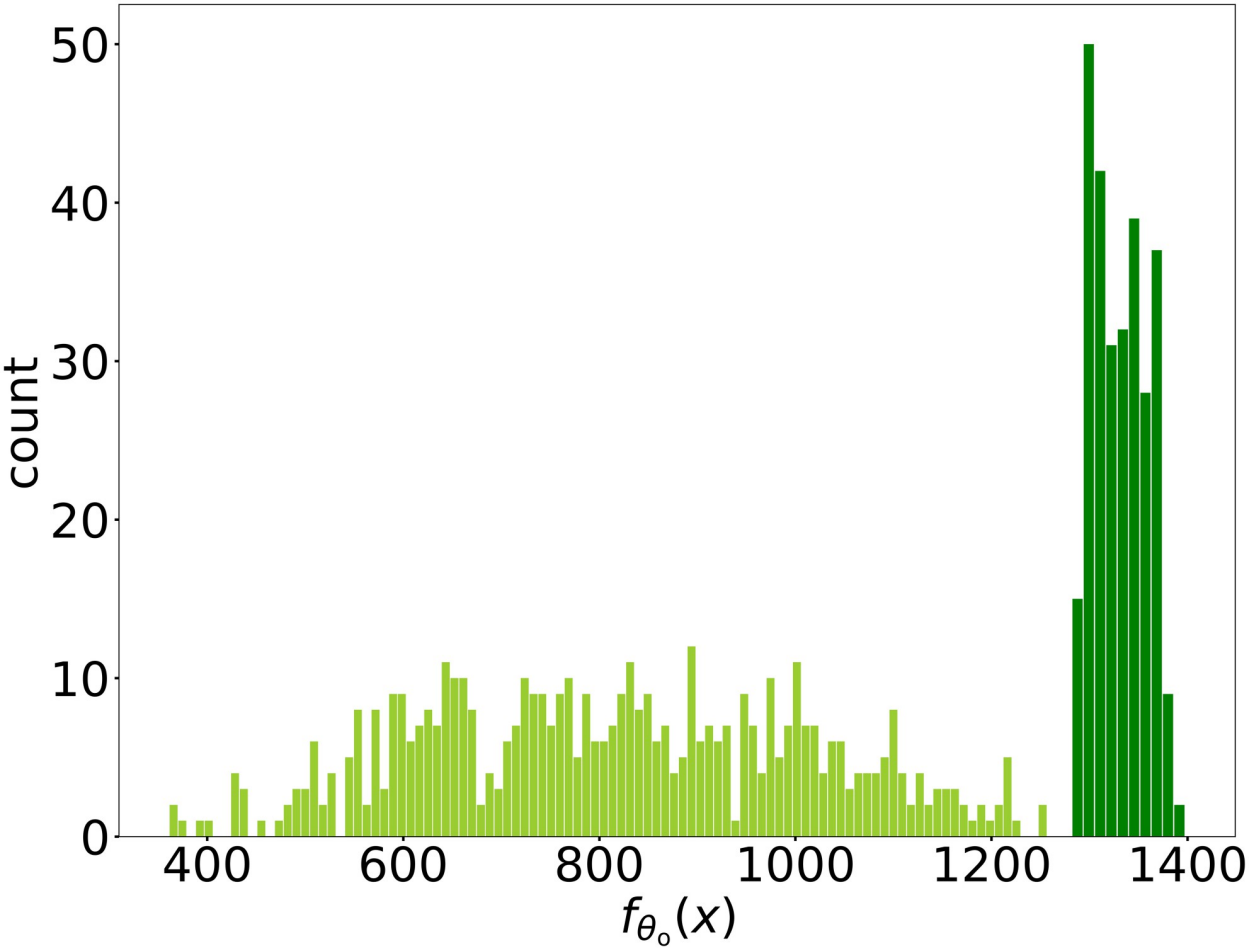
The distribution of the simulated actual flow value obtained by DiDo. The distribution of the simulated actual flow value on sampled data used for training DNN surrogate and the candidates of optimal parameters obtained by DiDo finally. The light green bars correspond to the training samples and the dark green bars correspond to the candidates of optimal parameters. The simulated actual flow values of all candidates obtained by DiDo are greater than the largest simulated actual flow value in the training data, demonstrating the effectiveness of our DiDo approach.

The optimal of this optimization problem may be not unique and there could be multiple local minima. Therefore, we solve the problem by gradient descent with various initial points to search for a global minimum. For visualization, in Fig 5, we show the distribution of the actual flow of the training samples used for learning DNN surrogate and a set of true feasible candidates of optimal profile parameters. Note that the maximal actual flow of training samples approximates 1256. After solving the optimization problem, we obtain a set of candidates of optimal profile parameters. Then we examine whether those parameters are in true feasible region with simulator and calculate the actual flow on these feasible designed rotor parameters with CFD simulator. The best actual flow we achieved is roughly 1400, which is better than those obtained by manually tuning parameters and the maximal actual flow of training samples 1256. The candidates of optimal profile parameters outperform the training samples in the sense of the actual flow. Most of the actual flow of the candidates of optimal profile parameters are larger than 1340.

**Fig 6:**
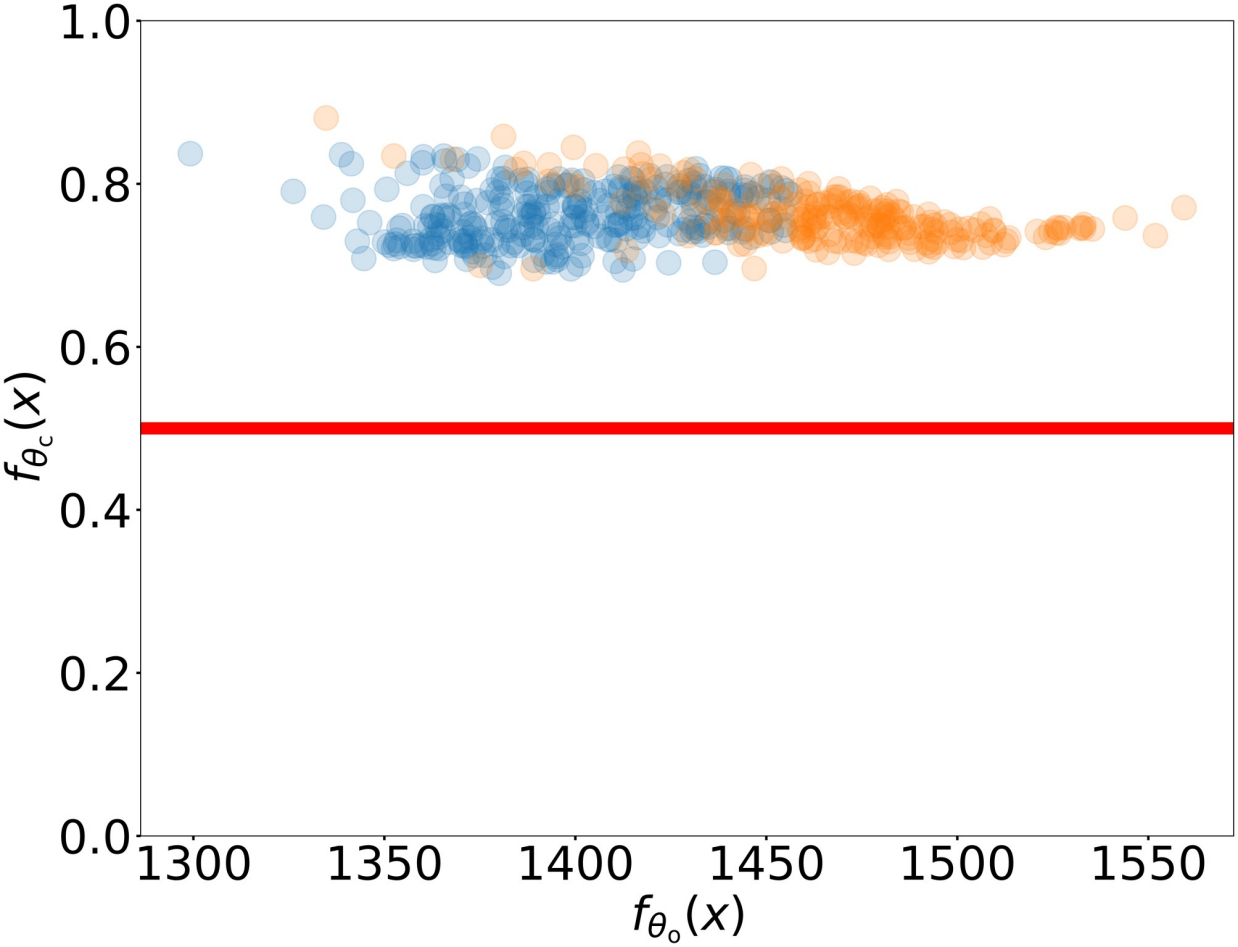
Property of candidates of optimal parameters for rotor profile design. The classifier value $f_{\theta_{c}(x)}$ and the actual flow predicted by DNN surrogate $f_{\theta_{o}(x)}$ on these candidates of optimal parameters. The red solid line is corresponding to the probability 0.5. Both blue and yellow dots are feasible predicted by DNN, both above the solid red line. However the yellow points are outside the true boundary. Therefore candidates of optimal parameters are close to boundary of true feasible region, signifying the importance of a highly accurate surrogate of the feasible region as obtained by our DNN-based adaptive fitting approach.

Further more, it is interesting to analyze the candidates of optimal parameters obtained using our DiDo approach. We analyze the distance between the candidates of optimal parameters and the boundary of the feasible region by computing the probability predicted by the classifier $f_{\theta_{c}}\left(x\right)$. As shown in Fig 6, each point corresponds to a candidates of optimal parameter and the $f_{\theta_{c}}\left(x\right)$ of obtained candidates of optimal parameters significantly deviate from 1, i.e., most candidates of optimal parameters with different actual flow predicted by DNN surrogate (abscissa) are close to the surrogate boundary (ordinate). Moreover, many of candidates are outside true feasible region examined by the simulator, i.e., these candidates are falsely classified as feasible ones by neural network (see yellow dots in Fig 6). Therefore these candidates are close to the true boundary. For such a problem, obtaining an accurate surrogate classifier is key to our optimization. Therefore, our DNN-based adaptive fitting approach, which can adaptively improve the accuracy of the DNN classifier, is a key procedure for a good performance of our DiDo approach.

## 5 Toy example: Harmonic function

To verify the validity of our method in solving high-dimensional data-informed optimization problem. Inspired by the practical problem of the rotor design, we construct a 100-dimensional optimization problem, whose optimal points locates on the boundary of the feasible region.

### 5.1 Problem description

We consider an optimization problem, where the objective function *f*(***x***) is a harmonic function $f\left(x\right)=-\left(x_{1}^{2}-\frac{1}{d-1}\sum_{i=2}^{d}x_{i}^{2}\right)$, which satisfies Poisson’s equation ∇^2^ *f*(***x***) = 0. The feasible region is $\Omega =\left\{x|||x||\leq 1\right\}=\left\{x={\left(x_{1},...,x_{d}\right)}^{T}|\sqrt{\sum_{i=1}^{d}x_{i}^{2}}\leq 1\right\}$.

The toy optimization problem is as follows,

$$\begin{array}{c} \underset{x}{\text{min}}\;-\left(x_{1}^{2}-\frac{1}{d-1}\sum_{i=2}^{d}x_{i}^{2}\right)\\ \;s.t.\;\sqrt{\sum_{i=1}^{d}x_{i}^{2}}\leq 1 \end{array}$$ (4)

where $x={\left(x_{1},...,x_{d}\right)}^{T}\in R^{d}$. For demonstration, we take *d* = 100.

Note that the harmonic function *f*(***x***) satisfies extremum principle, which indicates that the minimum of problem (4) is achieved on the boundary. As for the given case, it is clear that the minimum −1 is obtained at ***x*** = (1, 0, …, 0)^*T*^ and ***x*** = (−1, 0, …, 0)^*T*^. Remark that although the objective function and the constraints are analytically known, we assume that the objective function and the constraint functions can only be measured through sampling.

### 5.2 Feasible region learned by a DNN classifier

Similarly to the rotor problem, with the same settings, we first train a DNN classifier to learn the feasible region. We set initial sample size *n*_0_ = 3000, initial sample region *B* = [−0.173, 0.173]^100^ and we set *n*_1_ = 5000 samples in each iteration. By algorithm 2, we obtain a well-trained classifier $f_{\theta_{c}}\left(x\right)\in \left[0,1\right]$. We use the surrogate feasible region $\left\{x|f_{\theta_{c}}\left(x\right)\geq 0.5\right\}$ to represent the true feasible region Ω.

**Fig 7:**
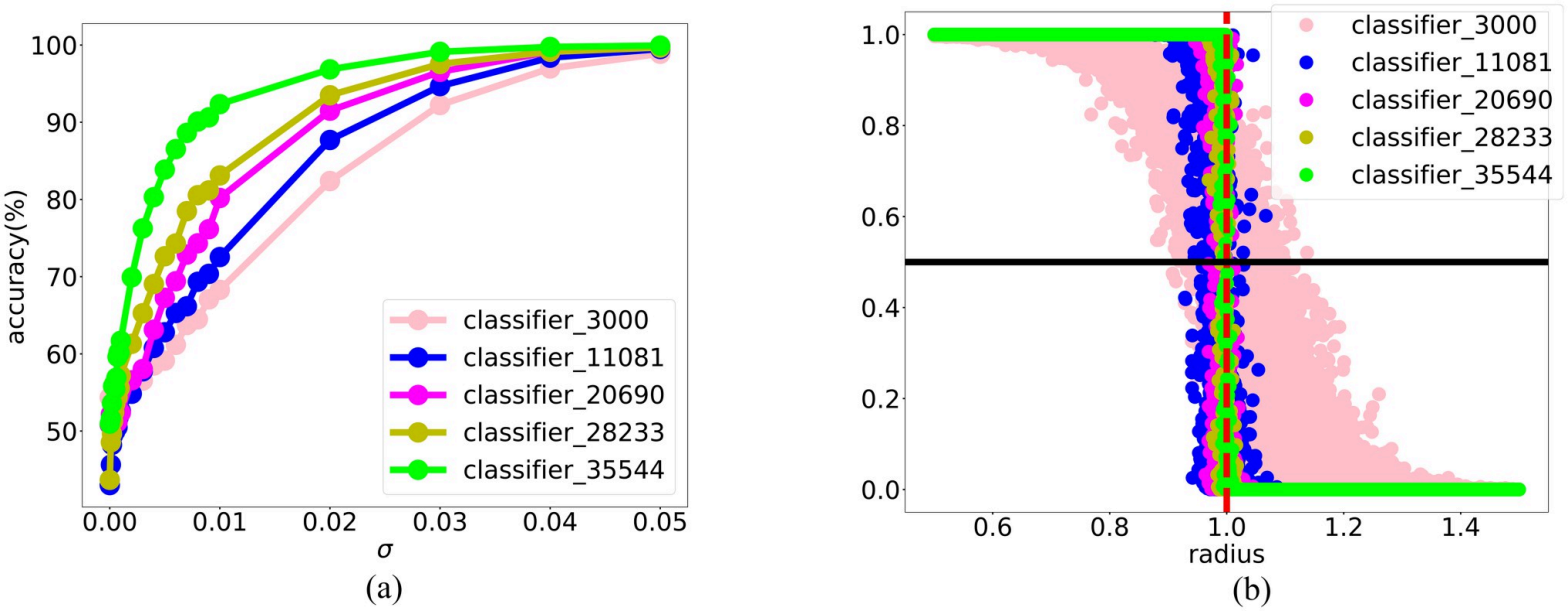
The improvement of DNN classifier through adaptive fitting. (a) Classification accuracy of the DNN classifier on the perturbed terms during iteration. Note that, there are not all iteration results and at each iteration *t*, we apply an extra constraint $|f_{\theta_{c}}^{\left(t\right)}\left(x_{i}\right)-0.5\left|\leq 0.1\right\}$ to the points sampled by LMC. In the two figures, label accuracy means classification accuracy after perturbation. As we add more data, the magnitude of the perturbed term when classifier accuracy on perturbed term increase from 50% sharply gets smaller, which means the distance between the true boundary and surrogate boundary gets smaller, i.e., the performance of classifier is better; (b) The classifier values on the points uniformly distributed along the radial direction. As the iteration proceeds, the classifier is more closed to the real classification function *I*(*r* ≤ 1).

During the DNN-based adaptive fitting, the accuracy of the classifier with Gaussian noise perturbation efficiently improves as shown in Fig 7(a). In addition, for this toy example, we know the real feasible region is a unit ball and it is clear to visualize the boundary along the radial direction. Thus, we calculate $f_{\theta_{c}}\left(rx\right)$, where ***x*** is uniformly sampled on the real boundary of the feasible region and *r* follows uniform distribution on the interval [0, 2]. As shown in Fig 7(b), throughout the DNN-based adaptive fitting, the surrogate classifier approximates the true feasible region *I*(*r* ≤ 1) better and better.

### 5.3 Objective function learned by a DNN model

**Fig 8:**
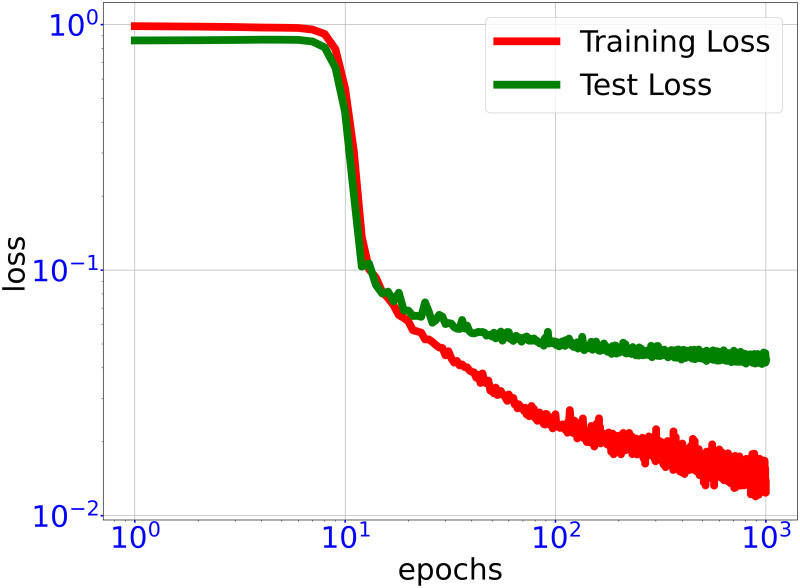
Training trajectory of DNN loss functions. Trajectory of training loss and test loss through training DNN surrogate $f_{\theta_{\text{o}}}\left(x\right)$ for fitting objective function of 100-dimensional toy optimization problem. In the end of training, the test loss is significantly larger than the training loss, indicating the DNN training is close to convergence.

By algorithm 3, we use the classifier $f_{\theta_{c}}\left(x\right)$ obtained above to generate a training set *D*_obj_ of size *n*_*t*′_ = 5, 000 and train a GELU-DNN $f_{\theta_{\text{o}}}\left(x\right)$ of hidden layer size 2000-1000-600-400-200. Remark that in algorithm 3, the ratio of the data in true feasible region to the data obtained by LMC is close to 1 since the classfier is about accurate. The test accuracy of the DNN $f_{\theta_{\text{o}}}\left(x\right)$ is evaluated on a test set consisting of 2000 samples. As shown in Fig 8, after training, the normalized RMSE training error is ∼0.01 whereas the normalized RMSE test error is ∼0.04.

### 5.4 Deep optimization

**Fig 9:**
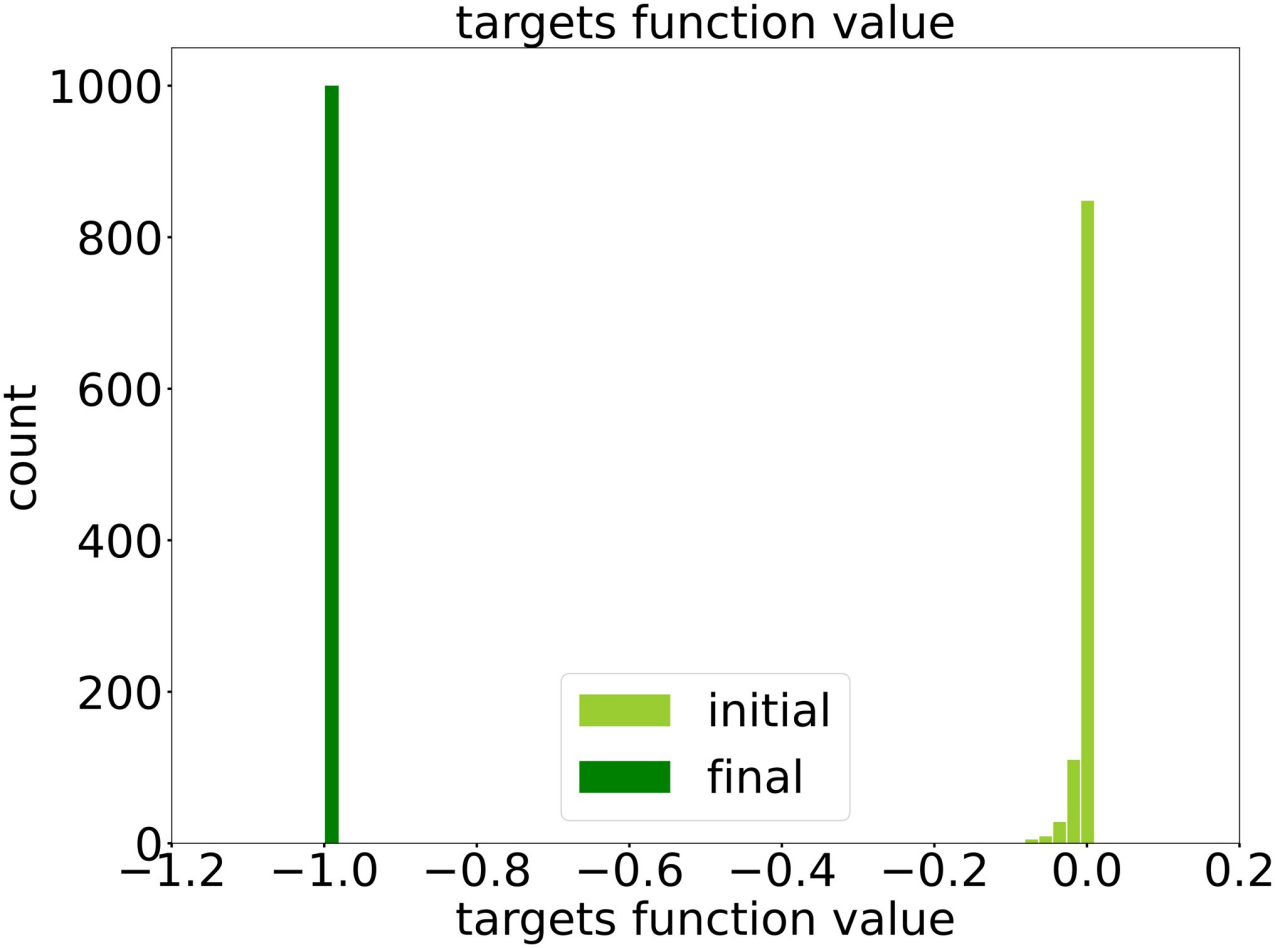
The distribution of the objective function values obtained by DiDo. Comparison between the objective function values on the initial points, i.e., the training samples used for learning DNN surrogate, and that on final candidates of optimal parameters. The minimum objective function value among training samples used for learning DNN surrogate ∼−0.1, whereas the objective function values of the candidates of optimal parameters concentrate around −0.98 very close to the true minimum −1 of this problem.

With DNN surrogate $f_{\theta_{\text{o}}}\left(x\right)$ and the well-trained DNN classifier $f_{\theta_{c}}\left(x\right)$, by algorithm 4, we obtain a set of candidates from different initial points. Note that we set the training samples used for learning DNN surrogate as initial points. For visualization, in Fig 9, we show the distribution of the objective function values of the initial points as well as that of the corresponding candidates of optimal parameters. Note that the minimum objective function value among training samples used for learning DNN surrogate ∼−0.1, whereas the objective function values of the candidates of optimal parameters concentrate around −0.98 very close to the true minimum −1 of this problem.

## 6 Conclusion and discussion

In this paper, we explore the application of DNN to solve a specific class of data-informed optimization problems with implicit constraints and objective function, emphasizing on DNN-based adaptive fitting approach to deal with potentially high-dimensional and complex constraints. Our results reaffirm deep learning as the key technique to solve high-dimensional problems suffering from the curse of dimensionality for both sampling and fitting. Moreover, our work shows that adapting to the problem is the key to the design of a DNN-based method. It is hoped that our DiDo approach not only help solve the data-informed optimization problems, but also inspire the future design of DNN-based algorithms in face of the curse of dimensionality for sampling.

For a type of high dimensional optimization problems, whose optimal points located in the interior region, e.g., maximize the Gaussian function in a unit ball, we find that it is more difficult to sample sufficient useful points to fit the objective function well. This phenomenon is due to the concentration phenomena in high dimension space [47]. For example, if we uniformly sample data in an unit ball, the samples concentrate at an O(1/d) shell of the surface. In practice, this phenomenon can be alleviated by using a proper sampling distribution, say radial uniform sampling, according to prior knowledge.

In the DNN-based adaptive fitting process, the hyperparameter *β* in LMC is important to sample diverse points close to the surrogate boundary. If *β* is too large, we observe that the added points concentrate at the surrogate decision boundary and the new classifier can even become less accurate. This phenomenon is related to frequency principle, i.e., the points close to the boundary are high frequency in nature, thus may result in worse generalization performance [34]. Empirically, proper *β* is needed for a steady improvement of accuracy of the DNN classifier.

Our DiDo approach as a data-driven approach requires sufficient data to obtain accurate surrogates for both constraints and objective functions which can be expensive for certain real application problems. We have demonstrated that DiDo with adaptive sampling can help overcome the curse of dimensionality in sampling, however, how much it can help in practice to improve the state of the art design in industrial problems remains to be evaluated due to the lack of benchmark [56].

In the future work, it is important to further improve the data efficiency of our approach, i.e. obtaining more accurate DNN surrogates with less sampling data. An important direction for the future study is to incorporate the structural information, such as duality, symmetry, conservation, etc, into the design of neural network methods for specific problems and application scenarios. The more structural information is incorporated, the less data is required for an accurate DNN fitting. Moreover, developing benchmarks for evaluation of the optimization methods for industrial problems is highly demanded. We will strengthen cooperation with the industry to gradually improve the situation.

## Supporting information

S1 Data (ZIP)
